# Development of a Piglet Grimace Scale to Evaluate Piglet Pain Using Facial Expressions Following Castration and Tail Docking: A Pilot Study

**DOI:** 10.3389/fvets.2017.00051

**Published:** 2017-04-18

**Authors:** Abbie V. Viscardi, Michelle Hunniford, Penny Lawlis, Matthew Leach, Patricia V. Turner

**Affiliations:** ^1^Department of Pathobiology, University of Guelph, Guelph, ON, Canada; ^2^Department of Animal Biosciences, University of Guelph, Guelph, ON, Canada; ^3^Ontario Ministry of Agriculture, Food, and Rural Affairs, Guelph, ON, Canada; ^4^School of Agriculture, Food, and Rural Development, Newcastle University, Newcastle upon Tyne, UK

**Keywords:** analgesia, animal welfare, grimace scale, piglet, pain, refinement

## Abstract

Facial expressions are increasingly being used to assess pain in non-human species, including rodents, horses, and lambs. The development of these species-specific grimace scales has allowed for more rapid pain detection, which can lead to better animal welfare if intervention promptly occurs. For grimace scales to ever be used as a stand-alone measure of pain, it is important they correlate with established pain assessment tools, such as behavioral analysis. This preliminary study aimed to determine whether piglets exhibit pain grimacing and if these facial expressions correlate with their behavior. It also assessed and compared the behavior of boar piglets given an analgesic and topical anesthetic prior to surgical castration and tail docking to piglets that did not receive anything for pain relief. Five-day-old male Yorkshire piglets (*n* = 19) from four pens were randomly assigned, within their pen, to one of five possible treatments: meloxicam (0.4 mg/kg, intramuscularly) + EMLA^®^ cream, meloxicam (0.4 mg/kg, intramuscularly) + non-medicated cream, saline (intramuscularly) + EMLA^®^ cream, saline (intramuscularly) + non-medicated cream, or no treatment prior to surgical castration and tail docking. Piglet behaviors were video recorded for 8 h immediately after castration, as well as for 1 h at 24 h pre- and post-castration. Their individual behaviors were scored continuously for the first 15 min of every hour of video collected. Facial images were also captured across all time points. A Piglet Grimace Scale (PGS) was developed and used by two observers blinded to treatment, time, and procedure to score over 600 piglet faces. All piglets displayed significant behavioral changes up to 7 h post-castration when compared to baseline, and the use of meloxicam and EMLA^®^ cream was not associated with a reduction in painful behaviors. Significantly higher PGS scores were noted at 0, 3, 4, and 5 h post-castration when compared to PGS scores at 7 h and there was no effect of treatment. PGS scores significantly correlated with piglet behavioral activity. The results suggest that the PGS may have utility for pain evaluation in neonatal pigs.

## Introduction

Facial expression analysis is widely used to assess pain in non-verbal humans (1). It has only recently been validated as a tool to evaluate pain in animals, with the development of species-specific grimace scales. Since the first grimace scale was introduced for mice in 2010, there have been scales developed for rats, rabbits, horses, sheep, and lambs (2–7). Grimace scales require identification of specific facial action units (FAUs) that change when animals are in pain, such as ear position, orbital tightening, and nose bulge. At least four FAUs have been described for each scale and all scales have demonstrated high inter-observer reliability among participants, suggesting they are accurate and easy to use. Their potential clinical application and ability to permit rapid pen-side detection of pain have generated interest in scale development for other species and further refinement of those that are pre-existing (8, 9). However, there are ethical issues with subjecting animals to unnecessary painful procedures for the development of a grimace scale.

Surgical castration and tail docking are routinely performed on commercially raised piglets in North America to reduce boar taint (an unpleasant odor and flavor associated with pork from intact males), aggression, and tail biting (10, 11). These procedures are known to cause acute pain persisting beyond 24 h, as indicated by behavioral and physiologic changes in piglets, including increased blood cortisol concentrations, high-frequency vocalizations, and trembling (10, 12–14). Behavioral analysis is often regarded as the gold standard for pain evaluation in animals (15), yet it is extremely laborious and impractical on a large-scale commercial farm. A grimace scale for piglets, if correlated with pain-related behaviors, would have great utility for on-farm assessments for its speed and ease of use. To determine whether these correspond, piglet behaviors and facial expressions before and after a known painful event (such as castration and tail docking) would need to be evaluated.

Castration and tail docking of piglets are often done without the use of an analgesic or anesthetic agent for pain relief. This may be due to drug approval limitations for food animals, or the added time, cost and effort involved with implementing analgesia into routine practice (16). There are analgesics licensed for use in piglets in North America and the European Union, including meloxicam, a non-steroidal, anti-inflammatory drug, which has pharmacologic effects lasting for at least 4 h (17). It has been shown to be efficacious in mitigating pain for procedures such as dehorning in calves as well as castration and tail docking in lambs (18, 19). Meloxicam has been used in previous piglet castration studies with varying results (14, 17). EMLA^®^ (Eutectic Mixture of Local Anesthetics) cream is a mixture of 2.5% lidocaine and 2.5% prilocaine that works as a topical anesthetic (4). It hasn’t been used on piglets, but it has been shown to reduce the pain of a scrotal injection prior to vasectomy in humans and tattooing in rabbits (4, 20). Legislation in Canada now mandates that analgesia be provided to piglets prior to surgical castration and tail docking (21) and there is an urgency to identify appropriate and effective analgesics and anesthetics to relieve postsurgical piglet pain.

The objectives of this preliminary study were to develop a pain scoring scale for piglets based on their facial expressions and to assess the effectiveness of 0.4 mg/kg of meloxicam and EMLA^®^ cream (individually and in combination) in reducing pain behaviors and facial grimacing of piglets following castration and tail docking. A decrease in pain behaviors and facial grimacing was expected in piglets receiving both the meloxicam injection and EMLA^®^ cream compared with piglets that did not receive any form of analgesic or anesthetic.

## Animals and Methods

### Animals and Treatments

Nineteen 5-day-old Yorkshire piglets from four different litters, weighing between 1.02 and 3.20 kg, were used. Sows and piglets were housed in farrowing pens at the University of Guelph Arkell Swine Research Station (Arkell, ON, Canada). The floor space for each pen was 6 ft × 8 ft (1.8 m × 2.4 m) and the farrowing crate was 2.5 ft × 7.5 ft (0.8 m × 2.3 m). The farrowing rooms were maintained at ambient temperature (23°C ± 0.5°C) with lights on/off at 07:00/21:00, and natural light was provided by windows in each room. Sows were fed *ad lib* 4 days after farrowing. The creep areas for piglets were heated to approximately 30–35°C by means of a heat lamp.

Five treatments were used and each treatment group was identified by a pre-determined symbol that was marked on the piglet’s forehead and back prior to castration. This was to keep individuals performing castrations and those involved in post-castration observations and behavioral scoring blinded as to animal treatment. Within each pen, piglets were randomly assigned to a treatment group (Table 1), and all treatments were represented in each litter (except for those that only had four boars). Meloxicam (Metacam 20 mg/mL; Boehringer Ingelheim Ltd., Burlington, ON, Canada) was administered at 0.4 mg/kg (liquid volume range of 0.1–0.32 mL) as an intramuscular injection. Saline was given intramuscularly at 0.2 mL. Then, 1.0 g of EMLA^®^ cream (EMLA^®^; Oak Pharmaceuticals Inc., Lake Forest, IL, USA; extra-label use) or 1.0 g of a sterile, non-medicated ointment (Life Brand Personal Lubricant Jelly; Shoppers Drug Mart Inc., Guelph, ON, Canada) was applied topically to cover the entire scrotal surface. The treatment groups were meloxicam + EMLA^®^ cream, meloxicam + non-medicated cream, saline + EMLA^®^ cream, saline + non-medicated cream, and no treatment (where no injection or cream was applied to the piglets prior to surgical castration and tail docking).

**Table 1:** **Number of piglets per pen assigned to each treatment group**.

Treatments	Pen A	Pen B	Pen C	Pen D
MEL + EMLA (meloxicam + EMLA^®^ cream)	1	1	1	2
MEL + 0 (meloxicam + non-medicated cream)	1^a^	1	1	1
SAL + EMLA (saline + EMLA^®^ cream)	1	1	1	0
SAL + 0 (saline + non-medicated cream)	1	1	1	2
None (no treatment)	0	0	1	1

*^a^Piglet was euthanized 5 h post-castration*.

### Processing Procedures

Twenty-four hours prior to study initiation, piglets were weighed and the meloxicam dose was calculated. On the day of the procedure, boar piglets were separated from their littermates, marked with a symbol using a black marker, and their assigned treatments were applied. Piglets were dosed at slightly more than 0.4 mg/kg meloxicam to account for the expected weight gain over the past 24 h. Approximately 20 min later, piglets were surgically castrated using a horizontal incision and tearing of the spermatic cord (16, 22). All piglet castrations occurred between 7 a.m. and 8 a.m. on the same day.

At the time of castration, the piglets also had their tails docked using blunt trauma cutters and were given iron intramuscularly (Iron Dextran; Dominion Veterinary Laboratories Ltd., Winnipeg, MB, Canada). The piglets were then returned to their pens. One piglet in the meloxicam + non-medicated cream treatment group was euthanized 5 h post-castration because of intestinal herniation through the castration site; all other piglets (*n* = 18) recovered from surgery without incident.

### Behavioral Recording and Scoring

Piglets were video recorded 24 h prior to castration for 1 h using a high definition video camera (JVC GZ-E200 full HD Everio Camcorder, Yokohama, Japan) mounted on a tripod. On the day of castration, the cameras were turned on immediately post-procedure and recorded for 8 h continuously. Finally, 24 h post-procedure, the cameras were turned on for 1 h. The behavior of each piglet was scored continuously by a single experienced observer blinded as to piglet treatment and time point for the first 15 min of every hour using the Observer XT program (Version 9.0: Noldus Information Technology, Wageningen, Netherlands) according to an ethogram adapted from a previous study assessing castration pain in piglets (10) (Table 2). All video clips were randomized using a random number generator program (www.random.org) prior to being scored. Intra-scorer reliability was tested every 3–4 weeks by having the observer rescore a video they had previously scored. The intraclass correlation coefficient (ICC) was then determined to ensure behaviors were being scored consistently over time and no drift had occurred (all intra-observer reliability tests produced an ICC above 0.9). A total of 2,700 min were observed and scored for this study.

**Table 2:** **Ethogram for scoring piglet behavior, grouped into feeding, locomotion, non-specific behaviors, pain-related behaviors, posture, and social cohesion [adapted from Hay et al. (10)]**.

Behaviors	Description
Suckling	Teat in mouth and suckling movements
Nosing udder/looking for teat	Nose in contact with udder, up, and down head movements

Playing	Springing, bouncy movements with littermates
Agonistic	Biting or fighting other littermates
Walking	Moving forward at a normal pace
Running	Trot or gallop
Awake inactive	No special activity, but awake
Sleeping	Lying down, eyes closed

Nosing	Snout in contact with a substrate
Chewing	Nibbling at littermates or substrates
Licking	Rubbing the tongue over littermates, floor, or pen walls

Spasms	Quick and involuntary contractions of the muscles
Scratching	Rubbing the rump against the floor or pen walls
Tail wagging	Tail’s movements from side to side (or up and down)
Stiffness	Lying with extended and tensed legs
Trembling	Shivering, as with cold
Lying	Body weight supported by side or belly
Sitting	Body weight supported by hindquarters and front legs
Standing	Body weight supported by four legs
Kneeling	Body weight supported by front carpal joints and hind legs

Isolated	Alone or with one littermate at most, distance of 40 cm separates the animal(s) from the closest group of littermates
Desynchronized	Activity different from that of most littermates (at least 75%)

After the piglet behaviors were analyzed separately, they were grouped into “active” and “inactive” categories. Active behaviors included walking, playing, nosing, suckling, and running. Inactive behaviors included sleeping and awake inactive. Postures were used for this behavioral analysis; piglets that were standing or sitting were scored as performing an “active” behavior and piglets that were lying were scored as demonstrating an “inactive” behavior. The sitting posture was placed in the active behavior category because most of the piglets exhibited this posture when suckling or scratching and these were considered active behaviors.

### Piglet Grimace Scale (PGS) Recording and Scoring

The PGS was developed using still images of piglet faces taken from the raw video recordings used for behavior scoring. Videos were uploaded to the PlayMemories Home program (Sony Corporation, Tokyo, Japan, 2014) and whenever a piglet face or profile was in view, the video was paused and the still image was saved, omitting times when piglets were lying down to sleep (to prevent facial changes due to tiredness being misinterpreted as pain faces). Images were captured across all time points and treatments, for a total of 627 faces (Table 3). Prior to scoring, the images were imported into Photoshop (Adobe Systems Incorporated, CA, USA, 2014) and the symbol on the piglet’s forehead was blurred to ensure blind scoring.

**Table 3:** **Total number of facial images captured for Piglet Grimace Scale scoring**.

Time point (h)	Treatment					Total
	MEL + EMLA	MEL + 0	SAL + EMLA	SAL + 0	None	
−24	–	–	–	–	–	63
0	9	14	6	21	7	57
1	21	18	12	27	11	89
2	8	12	7	15	2	44
3	8	13	6	11	5	43
4	13	13	12	15	7	60
5	24	12	11	16	9	72
6	19	6	12	21	8	66
7	11	4	5	10	5	35
24	22	20	19	28	9	98
Total	135	112	90	164	63	627

**Figure 1:**
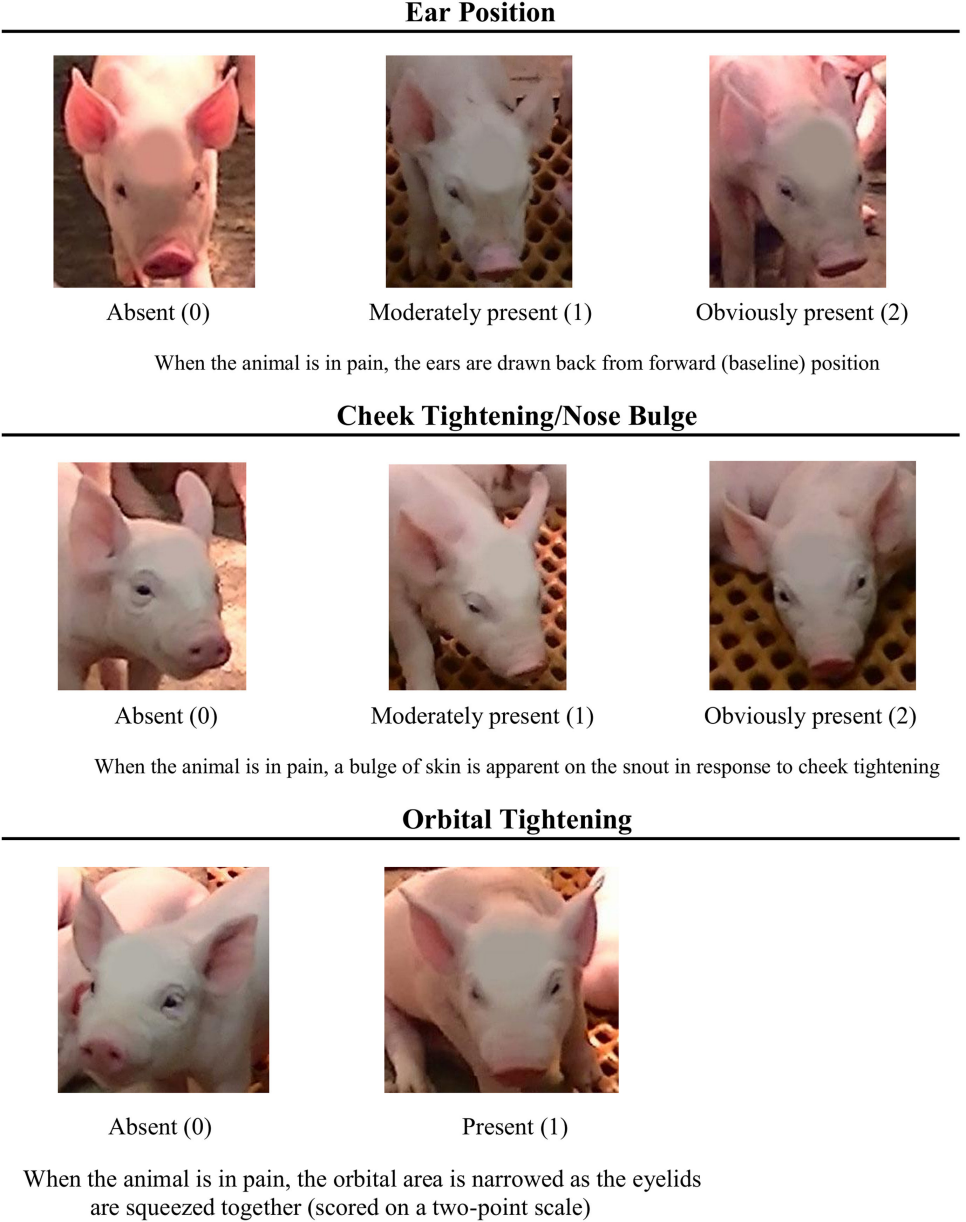
**Piglet Grimace Scale with descriptions for each of the three facial action units (FAUs) employed: ear position, cheek tightening/nose bulge, and orbital tightening**. FAUs are scored based on whether it is absent (score of 0), moderately present (score of 1), or obviously present (score of 2), with the exception of orbital tightening, which is scored on a 2-point scale of absent (score of 0) and present (score of 1).

To develop the PGS, images of piglet faces (across all treatments) were compared at different post-castration time points to the images pulled from the video recordings 24 h prior to castration (a time point that was assumed to represent a “no pain” state). Based on these comparisons, three significant FAUs were identified: orbital tightening, cheek tightening/nose bulge, and ear position (Figure 1), which are common to previously developed grimace scales. Cheek tightening/nose bulge and ear position were set to a 3-point scale (0–2), while orbital tightening was set to a 2-point scale (0–1). Therefore, the maximum pain score using the PGS was 5. Two individuals blinded as to piglet treatment and time point used the PGS to score the images. If an image could not be scored reliably, the observers were instructed to exclude it from scoring. The PGS score for each image was calculated by summing the scores allotted to each FAU. These “absolute” grimace scores were analyzed and then grouped into pain categories: scores of 0–1 represented a piglet experiencing “no-to-low pain” and scores of 3–5 represented “moderate-to-high pain.” A PGS score of 2 was excluded from these categories because of interpretation difficulty. The pain categories were transformed into proportions; at each time point, the proportion of piglet faces scored in the “no-to-low pain” category (low) and “moderate-to-high pain” category (high) was calculated. This was to allow correlation analysis between PGS score and active/inactive behavioral activity. A greater proportion of piglets falling in a “moderate-to-high pain” category would also give us information regarding analgesia efficacy, in that the drug (or dose) is insufficiently reducing pain. Appropriate analgesia would keep a majority of piglets in the “no-to-low pain” category.

### Data and Statistical Analysis

#### Behavior Analysis

The frequency and mean duration of the behaviors in Table 2 were square root transformed (except for awake inactive, sleeping, lying, and standing) to satisfy the assumptions of ANOVA (Statistical Analysis System 9.4, SAS Institute Incorporated, NC, USA, 2014). The normality of these behavior variables was tested using the univariate procedure prior to analysis. Data were analyzed using a mixed model ANOVA with piglet as the experimental unit. The model included pen, treatment, and time and the interaction between treatment and time. *Post hoc* tukey tests were conducted for significant factors with more than two levels using the least-squared means statement. The values presented here are the untransformed means with SE. Statistical significance was set at *p* < 0.05.

Durations of time spent active (standing + sitting) and inactive were converted to the proportion of total time observed; activity and inactivity were mutually exclusive. The proportions of activity/inactivity were then assessed to determine if they met the assumptions of ANOVA by testing that the residuals were normally distributed. The residuals of the raw proportions were normal (*W* = 0.99), so the proportions were not transformed. The proportion of activity and inactivity were analyzed as separate dependent variables using a mixed model analysis of variance; the model included treatment, time, treatment × time, and pen. LS means were calculated for all significant effects and a *post hoc* tukey adjustment was used. The values presented here are the untransformed means with SE. Statistical significance was set at *p* < 0.05.

#### Grimace Scale Analysis

The statistical analyses for the grimace scale scores were conducted using GraphPad Prism (GraphPad Software Incorporated, CA, USA, 2014). A two-way ANOVA was used to evaluate the scores provided for treatment and time and the interaction between them. A *post hoc* Tukey’s test was conducted for significant factors. SAS was used to determine the ICC of the two scorers. Both the behavioral data and the PGS data were used to determine if there was an effect of treatment in reducing the pain experienced by piglets undergoing castration and tail docking.

#### Correlation between Behavior and Grimace Scores

Correlation analyses were used to determine if there was an association between behavior and the PGS scores. The proportion of activity/inactivity over time and the proportion of high and low grimace scale scores were sorted by pen, treatment, and time. Residuals were analyzed and found to be normal (low: *W* = 0.98; high: *W* = 0.99; active: *W* = 0.98; inactive: *W* = 0.99), and the data were not transformed prior to analysis. The Correlation Procedure (SAS 9.4) and Pearson’s correlation coefficient were used to analyze the relationship between the proportion of time spent active or inactive with the proportion of low or high grimace scores. *R* and *p* values are presented.

## Results

### Behavioral Observations after Castration and Tail Docking

There were no litter-associated differences in behavior (*p* > 0.05) and data were combined across litters. Piglets demonstrated significant behavioral changes, when compared to baseline behaviors, up to 7 h post-castration and tail docking (for most behaviors, *p* < 0.0001). Only two behaviors, tail wag (*p* = 0.036) and isolated (*p* = 0.002), were affected by treatment across all time points, but overall, none of the treatments given to the piglets pre-castration and tail docking significantly reduced painful behaviors and postures (Table 4).

**Table 4:** **Behavioral analysis of castrated and tail docked piglets across all treatments and time points**.^d^

	Behavior	*F*_4,130_	p*	Treatment				
				None (19)^b^	MEL + 0 (37)	SAL + 0 (50)	SAL + EMLA (27)	MEL + EMLA (50)
Proportion (duration)	Playing	0.62	0.6508	0.90 ± 0.66	2.22 ± 1.25	0.85 ± 0.38	1.16 ± 0.61	0.81 ± 0.41
	Walking	1.34	0.2587	5.78 ± 2.33	9.24 ± 1.84	8.21 ± 1.74	8.91 ± 1.97	11.17 ± 2.06
	Running	0.56	0.6945	0.03 ± 0.02	0.01 ± 0.01	0.04 ± 0.04	0.07 ± 0.07	0.05 ± 0.03
	Awake inactive	1.36	0.2519	35.75 ± 5.5	45.60 ± 4.3	41.46 ± 3.95	51.47 ± 5.97	45.50 ± 3.82
	Sleeping	1.99	0.0997	57.47 ± 7.00	43.09 ± 5.79	49.25 ± 0.01	38.08 ± 7.30	42.27 ± 4.86
	Suckling	1.05	0.3861	7.83 ± 3.9	14.30 ± 3.35	13.50 ± 2.46	19.81 ± 5.30	15.61 ± 2.77
	Nosing udder	0.95	0.4355	1.64 ± 0.98	3.84 ± 1.16	7.15 ± 1.88	6.00 ± 1.65	6.02 ± 1.52
	Nosing	0.77	0.5474	5.94 ± 3.01	6.74 ± 1.81	5.67 ± 1.68	6.07 ± 1.62	7.18 ± 1.57
	Chewing	1.48	0.211	0.27 ± 0.13	1.23 ± 0.53	0.29 ± 0.14	0.47 ± 0.26	0.34 ± 0.14
	Stiffness	0.75	0.5614	0.93 ± 0.36	0.54 ± 0.13	0.67 ± 0.19	0.81 ± 0.26	0.78 ± 0.19
	Trembling	0.95	0.4373	0.50 ± 0.30	0.40 ± 0.26	0.04 ± 0.03	0.63 ± 0.34	0.85 ± 0.53
	Spasms	0.43	0.7845	1.12 ± 0.25	1.57 ± 0.77	0.90 ± 0.24	1.46 ± 0.53	1.12 ± 0.23
	Scratching	0.14	0.9675	0.38 ± 0.33	0.45 ± 0.27	0.29 ± 0.17	0.50 ± 0.36	0.33 ± 0.13
	Tail wagging	2.65	**0.036^c^**	0.94 ± 0.58	2.18 ± 0.81	0.60 ± 0.16	3.06 ± 1.01	2.68 ± 0.77
	Lying	0.94	0.4411	75.09 ± 7.19	66.67 ± 5.32	64.33 ± 4.93	64.68 ± 6.36	58.21 ± 4.85
	Sitting	1.17	0.327	4.13 ± 1.61	2.61 ± 0.57	2.95 ± 0.11	5.40 ± 1.80	4.89 ± 1.11
	Standing	1.99	0.0992	19.87 ± 6.70	30.3 ± 4.98	32.55 ± 4.77	29.47 ± 5.51	36.45 ± 4.64
	Kneeling	2.43	0.051	0.92 ± 0.49	0.69 ± 0.42	0.17 ± 0.11	0.44 ± 0.34	0.44 ± 0.22
	Isolated	4.67	**0.0015**	0.0 ± 0.0^a^	0.04 ± 0.04^a^	0.15 ± 0.15^a^	18.09 ± 11.59^a^	3.08 ± 2.50^a^
	Desynchronized	0.45	0.7717	1.94 ± 1.4	2.00 ± 1.68	1.56 ± 0.88	3.32 ± 2.36	1.92 ± 1.40

*^a^Statistical significance after *post hoc* Tukey test (*p* < 0.05)*.

*^b^Sample sizes for each treatment group*.

*^c^Interactions were not statistically significant after post hoc Tukey test*.

*^d^Twenty-four hour pre-castration for 1 h, 7 h immediately post-castration, and 24 h post-castration for 1 h*.

*Significant effects (*p* < 0.05) are indicated in bold.

**Figure 2:**
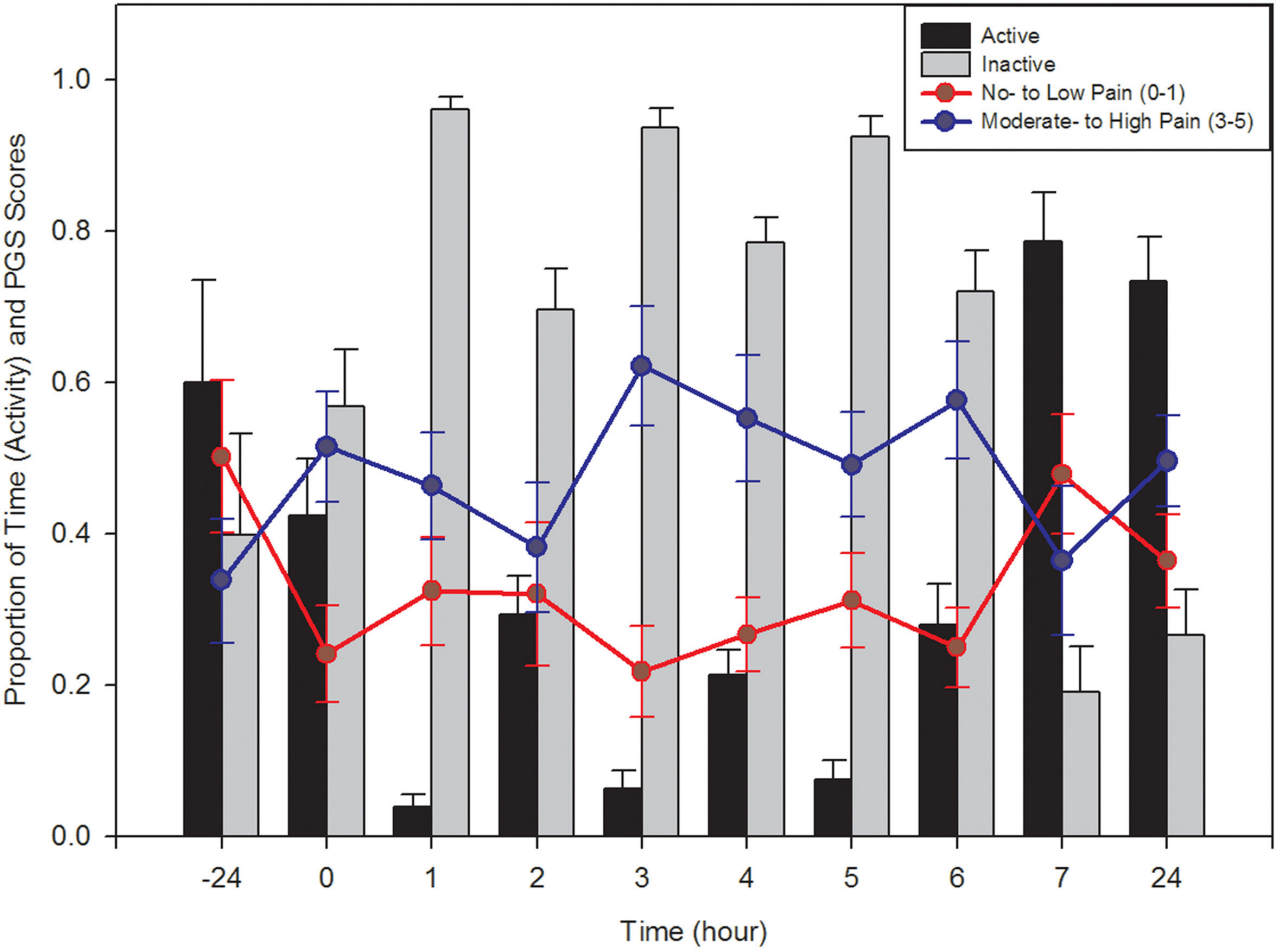
**Comparison of active (walking, playing, suckling, nosing, etc.) and inactive (lying, sleeping, isolated, awake inactive, etc.) behaviors of piglets at all time points, pre- and post-castration (±SE) are represented by the bar graph**. Active behaviors decreased significantly following castration, independent of treatment given, and eventually returned to baseline levels after approximately 7 h. Proportion (±SE) of Piglet Grimace Scale (PGS) scores within each pain category across all treatment types are represented by the line graph. Observers (*n* = 2) were unaware of piglet treatment or time point when scoring. PGS scores significantly correlated with piglet behavioral activity.

Active and inactive behaviors of piglets were also analyzed. Both behavior categories had a significant time effect (active: *F*_9,130_ = 25.75, *p* < 0.0001; inactive: *F*_9,130_ = 26.75, *p* < 0.0001), with piglets displaying more inactive behaviors than active behaviors up to 6 h post-castration and tail docking (Figure 2). There was no effect of treatment and no interaction between time and treatment (*p* > 0.05).

### Piglet Grimace Scale

Between-litter differences in facial grimacing were not noted and images were combined across litters. There was a significant treatment, time, and time × treatment interaction on PGS score (*p* = 0.005, *p* = 0.015, and *p* = 0.003, respectively). Further analysis found PGS scores at 0, 3, 4, and 5 h to be significantly increased than those at 7 h post-castration (*F*_4,15_ > 3.06, *p* < 0.05; Figure 2). There was no significant difference in pre-treatment PGS scores and those obtained 7 h after processing (*p* = 0.6404). PGS scores were significantly higher in piglets administered a saline injection and EMLA cream compared to piglets that were given a saline injection and non-medicated cream (*p* = 0.0083). Across all treatments, there was a similar pattern in PGS score, with the highest scores generally noted between 3 and 4 h post-procedure. There was moderate agreement between the scores of the two observers, with an ICC of 0.57.

### Correlation between Behavior and PGS

Piglet Grimace Scale scores and behavioral activity were strongly correlated (Figure 2). The proportion of low pain scores had a significant positive correlation with active behaviors (*r* = 0.222; *p* = 0.008) and a significant negative correlation with inactive behaviors (*r* = −0.218; *p* = 0.009). The proportion of high pain scores tended to negatively correlate with active behaviors (*r* = −0.159; *p* = 0.058) and positively correlate with inactive behaviors (*r* = 0.158; *p* = 0.061).

## Discussion

The results of this study suggest that 0.4 mg/kg of meloxicam and EMLA cream^®^ were unable to reduce piglet pain post-procedure. Meloxicam has been proven to be an effective analgesic for reducing postoperative pain in other food animal species, such as lambs and calves (18, 19), yet it has had limited success in significantly reducing castration pain in piglets (14, 16, 23). The dose of meloxicam used in this study (0.4 mg/kg) is consistent with current recommendations (24); however, this dose was not derived from analgesia studies in piglets. Rather, this dose has been shown to be effective when given one or more times to sows with mastitis–metritis–agalactia or when treating lameness in grower pigs (25). It may be that this study’s sample size was too small to evaluate treatment effects or that the dose of meloxicam provided to the piglets was not sufficient to reduce pain. EMLA^®^ cream also did not prove to be effective at providing topical anesthesia and reducing pain in this study. At the time of castration, piglets had their tails docked, yet EMLA^®^ cream was only placed on the scrotum. It may be that piglets were expressing pain behaviors (such as tail wagging) related to the tail docking procedure, for which they were given no local anesthetic (26). The duration of action for EMLA^®^ cream on human male genital skin is 15–30 min (27). Castration occurred 20 min after EMLA^®^ application, so it is possible that its full anesthetic potential was not reached. However, severing of the spermatic cord is known to be the most painful aspect of the castration procedure and EMLA^®^ cream’s anesthetic effects remain localized on the scrotum (28). It would not penetrate into the spermatic cord and is likely an impractical anesthetic to use to significantly reduce castration pain. A testicular injection of lidocaine may be better suited, but is not likely to be used in a commercial system because of the experience and time needed to administer the injection (29, 30).

This study found piglets in the saline and EMLA cream treatment group had the highest PGS scores compared to piglets administered saline and a non-medicated cream. The significance of this is difficult to interpret because of the low animal numbers in each group (3 and 5, respectively). Other studies using EMLA cream to reduce pain in rabbits, mice, rats, dogs, cats, and children have noted only mild irritation, if any, following topical use (4, 31–33). Future studies with larger piglet numbers are needed to evaluate the potential treatment effects on PGS score. Significant correlation was found between piglet behavioral activity and PGS scores; as piglet activity decreased, grimace scores increased and as piglet activity increased, grimace scores decreased. A higher grimace score indicates a greater pain state and a reduction in activity is consistent with pain expression (34). These results suggest that piglet grimaces do indicate pain and the PGS may be useful for future use in piglet pain assessment. Further, there was no difference in PGS score 24 h pre-procedure and 7 h post-procedure, indicating piglet’s facial expressions of pain are reduced to near the baseline level after 7 h (again, consistent with behaviors returning to baseline levels after 7 h). Other studies that have assessed piglets after surgical castration found behaviors indicative of pain persist from 2 h to 4 days (10, 12, 35, 36). This range in results could be because of differing castration techniques, piglet age, or overall study design variance.

Piglet Grimace Scale scores were grouped into pain categories to facilitate a direct comparison with piglet activity level. As this was a small-scale study, we felt it was important to ensure the scale works broadly before using it in large-scale studies, as data collection is quite time consuming. Grouping the PGS results also allows for easy interpretation of pain score. For example, piglets consistently falling in the “moderate-to-high pain” category are a more obvious cause for concern than piglets repeatedly scoring 3 or 4. Especially, if this is to be used on a production farm, by individuals with various levels of experience, it may be more clear and accessible to others to report the proportion of piglets in a pen that fall within each category of pain, than to report PGS numbers. The preliminary results of the PGS are promising and it will continue to be used and validated in future research.

The moderate inter-observer agreement found in this study could be a result of only identifying three FAUs for the PGS. The maximum pain score was significantly reduced from that reported in the other grimace scales (for example, the Mouse Grimace Scale had a maximum pain score of 10 versus 5 for the PGS) (2). It also may have been due to some of the images collected having low resolution, as faces were pulled from video data and not taken with a high-resolution camera. There was also no formal training session to teach the observers how to use the PGS, which may have accounted for the moderate inter-observer agreement. As this was a preliminary study, only two individuals were recruited to score piglet faces; future work will employ a larger group of scorers from different backgrounds to improve the validity of this scale.

A second grimace scale for piglets has been published recently by Di Giminiani et al. (37). The complexity of scales differ (10 FAUs were used in its development versus 3 FAUs in our scale) and orbital tightening was placed on a 3-point scale while we have orbital tightening set to a 2-point scale. Another study looking at piglet facial expressions used two FAUs only (orbital tightening and cheek tension) to assess pain (38). The decision to place orbital tightening on a “present” or “not present” scale and to limit the FAUs included (for example, collapsing cheek tightening and nose bulge into one FAU) in the current study was to ensure that this scale can be used easily and rapidly on-farm. Further validation is needed for both scales but having two available for piglets demonstrate the interest in grimace scale development and their importance as a tool for pain assessment. A limitation of this study is the small sample size of 19 piglets. While additional work is needed on a larger scale to further validate the PGS and confirm the lack of treatment effects, there have been grimace scales developed using less animals. The Lamb Grimace Scale was developed with 16 animals, although the authors do acknowledge that the results should be interpreted with caution due to these low numbers (6). Another study limitation is not having a strong behavioral baseline to compare to post-procedure behaviors. Therefore, it is unknown whether the increase in inactivity noted following castration was solely attributable to the surgical procedure versus partially attributable to normal circadian variation in activity levels of piglets. Future studies should assess baseline piglet behaviors at the same time of day as to minimize this possible confounding variable.

### Animal Welfare Implications and Conclusion

This preliminary study was able to demonstrate piglet grimacing in response to pain. It also confirmed that castration and tail docking cause significant pain in piglets lasting up to 7 h, as measured by detailed behavioral analyses and a newly conceived PGS. The current recommended dose of meloxicam (0.4 mg/kg) may not be sufficient at mitigating pain associated with castration and tail docking. The application of a local anesthetic (EMLA^®^) to the scrotum, with or without meloxicam, appears ineffective in reducing surgical castration pain. The PGS requires further validation but may become a useful tool to identify piglets experiencing acute pain, which will improve their welfare if prompt intervention occurs.

## Ethics Statement

All animal use and procedures were approved by the University of Guelph Animal Care Committee (Animal Utilization Protocol #3350). The institution is registered under the “Animals for Research Act” of Ontario and holds a Good Animal Practice certificate issued by the Canadian Council on Animal Care.

## Author Contributions

PT and PL conceived and designed the experiments. AV, PT, and PL conducted the studies and scoring. AV, MH, PT, and ML analyzed the data. AV, MH, and PT prepared the manuscript. All the authors edited the manuscript.

## Conflict of Interest Statement

The authors declare that the research was conducted in the absence of any commercial or financial relationships that could be construed as a potential conflict of interest.
